# Modulation of Lung Immune Response

**DOI:** 10.1155/2013/239020

**Published:** 2013-12-05

**Authors:** Alexandre de Paula Rogerio, Carlo José Freire Oliveira, Edinéia Lemos de Andrade, Oliver Haworth

**Affiliations:** ^1^Departamento de Clínica Medica, Laboratório de Imunofarmacologia Experimental (LIFE), Instituto de Ciências da Saúde, Universidade Federal do Triângulo Mineiro (UFTM), Rua Vigário Carlos 162, 38025-350 Uberaba, MG, Brazil; ^2^Instituto de Ciências Biológicas e Naturais, Universidade Federal do Triângulo Mineiro (UFTM), Uberaba, MG, Brazil; ^3^Universidade Federal de Santa Catarina (UFSC), Florianópolis, SC, Brazil; ^4^William Harvey Research Institute, Charterhouse Square, Queen Mary, University of London, UK

The knowledge of the participation of the innate and adaptive immune response on the pathophysiological process of infectious and noninfectious airway injuries is rapidly expanding and the advances in the fields of biomedicine and biotechnology have been decisive to this expansion. The innate and adaptive immune system, as well as structural cells, modulates the quantity and quality of airway inflammatory response. Aberrant immune responses, including those induced for allergens, environmental pollutants, infectious agents, acids, and others promote excessive leukocyte recruitment, production of proinflammatory cytokines, chemokines, and other immunomodulatory mediators which are critical to initiate and maintain airway disorders. In recent decades, considerable progress has been made in the understanding of the genetic and immunological factors that contribute to the development and/or treatment of airway disorders. Thus, the use of molecular and cellular assays together with knockout animals has contributed significantly to this evolution. In despite of this, the current therapy for the treatment of airway disorders has not changed to the same degree and is still far from ideal. This special issue covers the most relevant research regarding the immunological aspects of airways disorders including asthma, airway inflammation, cancer and fungal and viral infections. 

Allergic asthma is a complex inflammatory disorder characterized by airway hyperresponsiveness, eosinophilic inflammation, and hypersecretion of mucus. The asthma physiopathology involves chemical mediators and many cells, mainly eosinophils which plays an important role in inflammation establishment through the release of specific mediators. The effect of corticosteroids on the asthma treatment as well as their adverse effects is widely known. Montelukast (Singulair) is a cysteinyl-leukotriene [CysLT_1_]-receptor antagonist and demonstrates improvement of asthma; however the chronic use, also, causes adverse effects. It could be used in synergism with corticosteroids and in this case a lower dose of corticosteroids or both could be used and consequently less adverse effect could be evidenced. Using a classical airways allergic inflammation experimental model (induced by ovalbumin) N. B. Gobbato et al. demonstrated that either montelukast or dexamethasone was effective in reducing the recruitment of eosinophils to the lung and other parameters such as eotaxin, RANTES, and IGF-I concentrations possibly by interfering in the NF-*κ*B signaling pathway. These data suggest that both treatments contribute to a better control of the inflammatory response in airways. 

In the asthma, several cytokines, mainly Th2 cytokines, modulate and coordinate the airways immune response. L. Gallelli et al. reviewed the most current advances of anticytokine therapies in asthma, suggesting that antibodies anti-IL-4, anti-IL5, and anti-IL13 (all Th2 cytokines) may be beneficial in the treatment of atopic asthma and other allergic diseases but antibodies against cytokines such as IL-9, GM-CSF, TNF-*α*, IL-23, IL-17, IL-25, and IL-33 must also be considered. Regarding the role of cytokines in the lung immune response, IL-33 has been highlighted in recent years. IL-33 is a novel member of the IL-1 family. M. M. Bunting et al. demonstrated in an airways allergic inflammation experimental model (induced by ovalbumin) that the expression of IL-33 in the airways is enhanced. In addition, they demonstrated that neutralization of IL-33 (antibody anti-IL-33) decreased both airway inflammation and the expression of proinflammatory cytokines by airways macrophages. These results demonstrated that IL-33 could be an important target to the treatment of airways allergic inflammation.

Chemoattractant cytokines, named chemokines, are also involved in asthma pathogenesis. CXCL12 is a homeostatic CXC chemokine widely expressed with a broad range of functions (from recruitment of mature B and T cells to migration of haematopoietic progenitor cells from the bone marrow). H. Rafatpanah et al. analyzed for CXCL12 polymorphisms in asthmatic individuals and demonstrated polymorphisms at position +801 of CXCL12. Interestingly, the authors suggest that the systemic concentration of CXCL12 could be used as one of the important markers in asthma diagnosis. 

Carbon nanotubes (CNT) and carbon nanofibers (CNF) are particles present in an increasing number of consumer products and are also incidental components in indoor air. In an airways allergic inflammation experimental model (induced by ovalbumin), U. C. Nygaard et al. demonstrated that CNF and CNT (administrated by intranasal route) modulated the airway responses to allergens, resulting in airway inflammation and production of allergen-specific IgE. This study provided a basis for better understanding the mechanisms underlying to the effect of nanoparticles such as CNF and CNT promoting airway allergy. 

Oral tolerization with proteins modulates the immune response to promote attenuation of hypersensitivity reactions. Xavier-Elsas et al. using direct and indirect experimental model of tolerization (induced by ovalbumin) demonstrated that oral tolerization suppresses eosinopoiesis in the bone-marrow.

In the lung, the vagal parasympathetic nervous system via muscarinic receptors represents the autonomic control of airways muscle tone. Cholinergic system is involved in bronchoconstriction as well as smooth regulation of inflammation. In this context, Castro et al. evaluated the airways responses to the cholinergic agonist methacholine (MCh) in two mouse lines selected to respond maximally (AIRmax) or minimally (AIRmin) to an innate inflammatory stimuli, focusing mainly on the role of M2 receptors. In basal condition AIRmin mice responded more vigorously to MCh than AIRmax. Gallamine treatment, a specific M2 antagonist, increased airway response of AIRmax but not of AIRmin mice. The expression of M2 receptors in the lung was significantly lower in AIRmin when compared to AIRmax animals. AIRmax mice developed a more intense allergic inflammation than AIRmin and both allergic mouse lines increased airway responses to MCh. These authors suggested that AIRmin and AIRmax mouse lines represent a model to study lung muscarinic receptor functions and its relation with the development of an allergic lung disease.

 In chronic pulmonary disorders including cystic fibrosis (CF) and chronic obstructive pulmonary disease (COPD) neutrophils play an important role in airway inflammation by damaging the airways tissues. Secretory leukoprotease inhibitor (SLPI) is an anti-inflammatory protein present in respiratory secretions. The SLPI associated to neutrophils is still poorly characterized. In an elegant study, F. P. Reeves et al. demonstrated that recombinant human SLPI significantly inhibited fMet-Leu-Phe (fMLP) and interleukin (IL)-8 induced neutrophil chemotaxis as well as decreased degranulation of matrix metalloprotease-9 (MMP-9), hCAP-18, and myeloperoxidase (MPO) from neutrophils, probably due to modulation of cytosolic IP_3_ production and downstream Ca^2+^ influx. They suggest that SLPI could have potential therapeutic effects in neutrophilic airways diseases.

 Acute lung injury (ALI) and its severe form, acute respiratory distress syndrome (ARDS) are caused by several stimuli as direct (such as pneumonia, aspiration of gastric contents, and others) as indirect (such as sepsis, burns, pancreatitis, and others). No therapeutic agents have demonstrated a clear benefit in ALI treatment and corticosteroids have been used for the treatment of ALI for many years and present a range of side effects. D. C. Favarin et al. cover a wide range of studies, mainly in experimental model, in a review describing the natural compounds (extracts from medicinal plants and secondary metabolites isolated from them) with potential to be used as an alternative treatment for ALI. In another study demonstrated by S. F. Fernandez et al., airways macrophages exposures to a low pH (1.75) for 1 min resulted in reduction in all lipopolysaecharide (LPS) and lipoteichoic acid (LTA)-mediated cytokine release. In addition, they used a nonlethal experimental model of ALI from acid aspiration followed by pulmonary bacterial infection (*Escherichia coli* and *Streptococcus pneumonia*) and demonstrated reduction of bacterial clearance. They suggested a strong suppressive direct effect of low pH stress on the ability of alveolar macrophages to mount an adequate antibacterial proinflammatory response following TLR2 and TLR4 activation. 

Methamidophos, an organophosphate insecticide, is a well-known toxicant, and its use is restricted during planting of vegetables. W. Watanabe et al. evaluated the developmental immunotoxicity of methamidophos using a respiratory syncytial virus (RSV) infection mouse model. Pregnant mice were exposed to methamidophos and offspring born to these dams were intranasally infected with RSV. They demonstrated that methamidophos suppressed the production of proinflammatory cytokines in the bronchoalveolar lavage fluids and the inflammation in the lung in response to RSV infection in the offspring generation of mice. So, they suggested that exposure of the mother to methamidophos during pregnancy could cause an irregular immune response in the lung tissues in the offspring mice.

Bronchiectasis is an abnormal and irreversible thick-walled dilatation of the bronchi caused by the inflammatory response of the bronchi as consequence of recurrent lower respiratory tract infections. In case-control cross-sectional study, M. Luján et al. demonstrated that the prevalence of bronchiectasis was higher (20%) in the steroid-dependent asthma (SDA) group when compared to nonsteroid-dependent asthma (NSDA) group (4%). In addition, patients with asthma-associated bronchiectasis presented lower FEV_1_ (forced expiratory volume in one second) values than patients without bronchiectasis, while the levels of IgG immunoglobulins showed no differences between the tested groups. Therefore, steroid-dependent asthma group seems to be associated with a higher risk of developing bronchiectasis than nonsteroid-dependent asthma. 

Some individuals could be infected by *Aspergillus* moulds and can develop as airways as invasive diseases due to predisposing factors or defects in immune responses. In a review S. H. Chotirmall et al. covered the most important aspects involved in the airways infection caused by *Aspergillus* moulds such as clinical spectrum of disease, innate and inflammatory defenses against distinct *Aspergillus *species, and modulating the immune and inflammatory response for therapeutic purpose. In another article H. Sales-Campos et al. described the aspects immune response such as recognition, activation of innate and adaptive immune responses, pathogen virulence factors, and the development of novel therapeutic approaches for invasive lung aspergillosis.

Rheumatoid arthritis (RA) is a chronic, inflammatory condition that mainly affects joints and causes pain, erosion, and disability. In addition, interstitial lung disease (ILD) is a relevant extra-articular manifestation of RA that may occur either in early stages or as a complication of long-standing disease which was reviewed by L. Cavagna et al. The authors described the multifaceted aspects of interstitial lung disease in rheumatoid arthritis bringing important information in the field of epidemiology and prognosis, risk factors, histological and radiological patterns, and all the immunological involvement. 

A. Antczak et al. demonstrated that the polymorphism of cytotoxic T-lymphocyte-associated antigen-4 (CTLA-4), an immunoregulatory molecule that downregulates T-cell activation, is associated to nonsmall cell lung cancer (NSCLC). These authors showed that allelic and genotypic distribution of +49 A/G and −318 C/T CTLA-4 SNPs (single nucleotide polymorphisms) are present in a group of NSCLC patients and revealed that CTLA-4 genotype distribution shifts from one genotype in the blood to another genotype in the tumor.

